# Positive Affect and Negative Emotional Responses to Daily Stressors

**DOI:** 10.1093/geroni/igaa057.2189

**Published:** 2020-12-16

**Authors:** Kate Leger, Susan Charles, David Almeida

**Affiliations:** 1 University of Kentucky, Lexington, Kentucky, United States; 2 BSS, Irvine, California, United States; 3 Pennsylvania State University, University Park, Pennsylvania, United States

## Abstract

Positive affect is beneficial for regulating negative emotional responses to stressful events. Yet, few studies have examined if positive affect may attenuate negative affect the following day. We examined how both trait positive affect and state positive affect are associated with next day stressor-related negative emotions. Participants (N = 1,588) from the National Study of Daily Experiences II (NSDE II) and the Midlife in the United States survey (MIDUS II) answered questions about stressors and emotion across eight days. People high on trait positive affect reported less negative affect the day following a stressor. On days when people experienced a stressor and higher than average state positive affect, they experienced less negative emotion the following day. This held true regardless of whether people were high or low on trait positive affect. Positive affect can help explain both who and when people will have attenuated emotional responses to stressful events.

